# Limited prefrontal cortical regulation over the basolateral amygdala in adolescent rats

**DOI:** 10.1038/s41598-018-35649-0

**Published:** 2018-11-21

**Authors:** Ryan A. Selleck, Wei Zhang, Hannah D. Mercier, Mallika Padival, J. Amiel Rosenkranz

**Affiliations:** Cellular and Molecular Pharmacology, Center for Neurobiology of Stress Resilience and Psychiatric Disorders, The Chicago Medical School, Rosalind Franklin University of Medicine and Science, 3333 Green Bay Road, North Chicago, IL 60064 USA

**Keywords:** Weak Excitatory Effect, Summation Ratio, Stimulus Number, Typical Excitatory Response, Pulse Interaction, Amygdala, Neural circuits

## Abstract

Cognitive regulation of emotion develops from childhood into adulthood. This occurs in parallel with maturation of prefrontal cortical (PFC) regulation over the amygdala. The cellular substrates for this regulation may include PFC activation of inhibitory GABAergic elements in the amygdala. The purpose of this study was to determine whether PFC regulation over basolateral amygdala area (BLA) *in vivo* is immature in adolescence, and if this is due to immaturity of GABAergic elements or PFC excitatory inputs. Using *in vivo* extracellular electrophysiological recordings from anesthetized male rats we found that *in vivo* summation of PFC inputs to the BLA was less regulated by GABAergic inhibition in adolescents (postnatal day 39) than adults (postnatal day 72–75). In addition, stimulation of either prelimbic or infralimbic PFC evokes weaker inhibition over basal (BA) and lateral (LAT) nuclei of the BLA in adolescents. This was dictated by both weak recruitment of inhibition in LAT and weak excitatory effects of PFC in BA. The current results may contribute to differences in adolescent cognitive regulation of emotion. These findings identify specific elements that undergo adolescent maturation and may therefore be sensitive to environmental disruptions that increase risk for psychiatric disorders.

## Introduction

Mammalian adolescents exhibit less top-down regulation over behavior than their adult counterparts. This manifests as greater risk-taking behavior, impulsiveness, mood fluctuations and labile emotional responses^1–8^. The adolescent period is associated with anatomical and functional maturation of several key structures that produce and regulate mood and emotion, most notably, the prefrontal cortex (PFC) and the amygdala^9–11^. Human imaging studies indicate that the functional interaction between PFC and amygdala develops across adolescence, and shifts towards a negative relationship between PFC and amygdala activity, wherein elevated PFC activity is correlated with decreased amygdala activity during tasks that require cognitive appraisal or regulation of emotion^7,10,12–21^. Conditions that feature impaired regulation of emotion are associated with a deficit in the strength of this relationship between the PFC and amygdala^22–28^. The cellular substrates of this relationship are beginning to be uncovered.

The PFC sends robust glutamatergic projections to the basolateral amygdala (BLA) that can drive neuronal activity. But these projections can also regulate the amygdala through several inhibitory intermediates, such as activation of GABAergic interneurons in the BLA and in the intercalated cell masses^29–37^. This restrictive relationship between PFC and BLA activity can guide or regulate the expression of anxiety and fear. However, these PFC projections still undergo refinement in late adolescence^31,38^. Furthermore, weaker regulation of emotion in adolescents implies that the PFC does not exert a potent influence over the adolescent amygdala. The neurobiological substrates for this functional immaturity are not entirely clear, but might include weaker excitatory effects of PFC onto BLA principal output neurons or onto BLA inhibitory interneurons, or weaker inhibitory influences of these interneurons on BLA principal neurons. The only study to date that examined this^31^ found that PFC inputs to BA exert weaker direct excitatory effects in immature mice. While this was a very important finding, it focused only on a part of the BLA, and by necessity had technical limitations of the *in vitro* approach and optogenetic viral infections at different ages. The purpose of the current study was to measure *in vivo* excitatory and inhibitory effects of PFC inputs to BLA, and to test (1) whether the adolescent BLA *in vivo* is under less inhibitory regulation by the PFC than the adult BLA, and (2) if this weaker impact of PFC on BLA can be ascribed to differences in the excitatory or the inhibitory effects of PFC inputs.

The rat PFC includes the prelimbic (PrL) and infralimbic (IL) cortex. While both areas project across multiple nuclei of the BLA, the PrL projects more densely to the rat basal nucleus (BA) of the BLA whereas the IL projects more densely to the lateral nucleus (LAT)^39–41^. Both inputs can activate BLA interneurons^30,32^, but there is some inconsistency about whether PFC and other cortical inputs equally produce inhibition in LAT and BA^30,42–44^, perhaps due to use of a slice preparation that severs substantial local connectivity or differences in age. PrL and IL play complementary roles in BLA-dependent behaviors. It has been proposed that PrL plays a major role in guiding learned fear behaviors while IL plays a major role in extinction of learned fear^45–50^. Therefore, it is important to separately examine PrL and IL inputs to BA and LAT. An additional goal of this study was to clarify differences between adolescent and adult rats in the response to PrL and IL PFC regions *in vivo*.

## Results

### Impact of PFC inputs to the BLA

**Figure 1:**
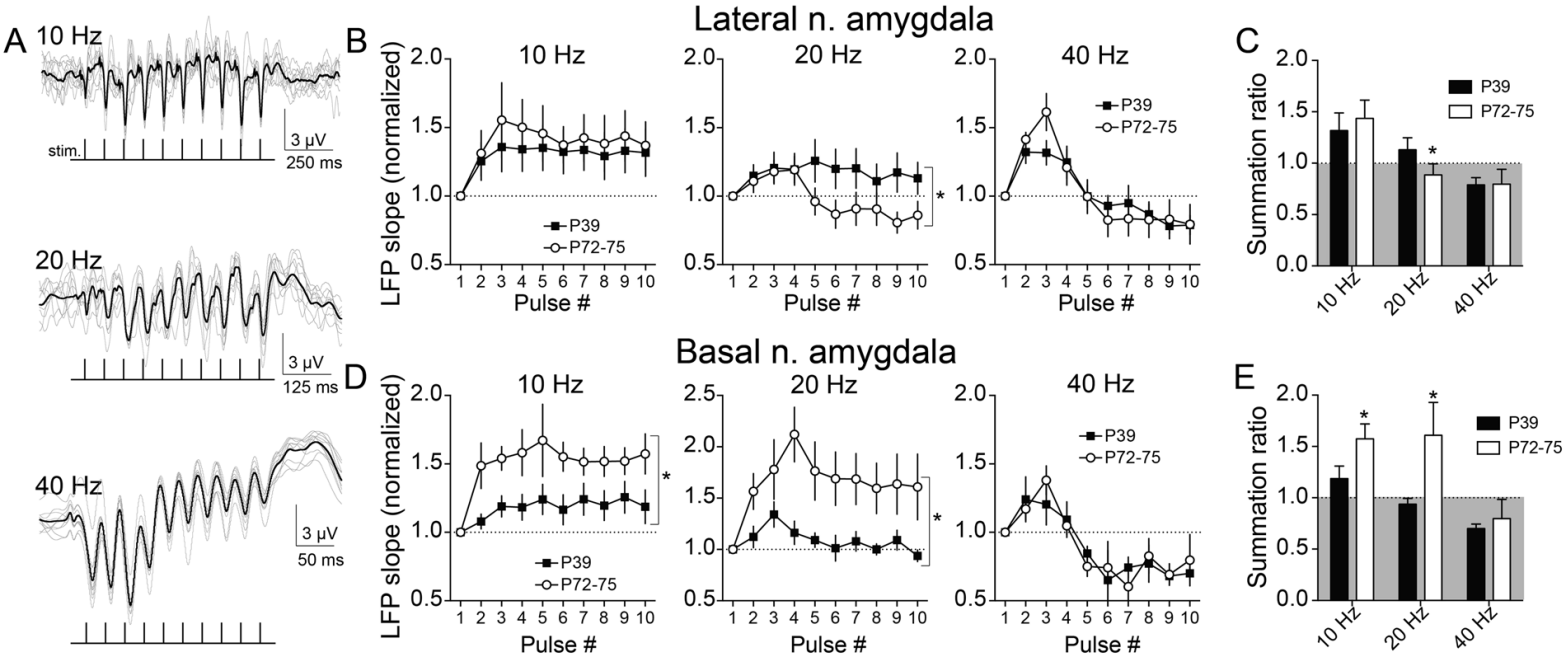
Age differences in summation of evoked field potentials in adolescent amygdala. The prefrontal cortex (PFC) was stimulated at different frequencies (10, 20, or 40 Hz, 10 pulses/train) while recording in the lateral (LAT) or basal (BA) nuclei of the amygdala. **(A)** Evoked local field potential (LFP) facilitation/depression depended on the train stimulation frequency, shown here as overlays of 10 consecutive responses (grey traces) and the overlaid average (black trace). **(B)** In the LAT, facilitation of LFPs at 20 Hz was suppressed in PND 72–75 rats (normalized value < 1.0) across the train, and significantly different than PND 39 rats (*p < 0.05, two-way ANOVA). In contrast LFPs showed similar facilitation between PND 72–75 and PND 39 at 10 Hz and similar depression at 40 Hz (p > 0.05, two-way ANOVA). **(C)** The summation ratio (last LFP/first LFP) was significantly lower in PND 72–75 rats at 20 Hz (*p < 0.05, post-hoc Holm-Sidak’s multiple comparisons test after two-way ANOVA). **(D)** In the BA, significant LFP facilitation was observed at 10 and 20 Hz, and this was greater in PND 72–75 compared to PND 39 rats (*p < 0.05, two-way ANOVA), while a similar depression was observed at 40 Hz (p > 0.05, two-way ANOVA). **(E**) The summation ratio was greater in PND 72–75 rats at 10 Hz and at 20 Hz compared to PND 39 rats (*p < 0.05, post-hoc Holm-Sidak’s multiple comparisons test after two-way ANOVA).

To gauge the BLA response to PFC inputs, LFPs in the BLA were measured during train stimulation of PFC and compared between adults (PND 72–75) and adolescents (PND 39). The LFP response in both LAT and BA were examined (n = 11 hemispheres from 11 rats from each age; Supplemental Figs 1 and 2). Three frequencies were chosen to roughly mirror three primary bands of cortical activity in awake animals (10 Hz, alpha/theta; 20 Hz, beta; 40 Hz, gamma; Fig. 1A). These same frequencies of interneuron activity produce contrasting recruitment of inhibition and short term dynamics of GABAergic systems in the BLA^51,52^. LFPs can increase across the train (facilitating) or can decrease across the train (depressing) depending on several factors, including interaction between evoked inhibitory and excitatory synaptic inputs. Facilitation was dependent upon stimulation frequency in LAT (PND 72–75: frequency x pulse interaction, p < 0.0001, F(18,261) = 5.332, n = 11 rats; PND 39: frequency x pulse interaction, p < 0.0001, F(18,270) = 3.899, n = 11 rats, two-way RM-ANOVA) and in BA (PND 72–75: frequency x pulse interaction, p < 0.0001, F(18,270) = 3.837, n = 11 rats; PND 39: frequency x pulse interaction, p = 0.0026, F(18,270) = 2.282, n = 11 rats, two-way RM-ANOVA). Facilitation (normalized LFP slope >1) was generally observed at 10 Hz (Fig. 1B,D, left) while depression (normalized LFP slope <1) was observed at 40 Hz (Fig. 1B,D, right). Depression at 40 Hz suggests that higher stimulation frequencies recruit activation of BLA inhibitory elements that suppress summation^53^.

To determine whether LFP facilitation shifted from adolescence to adulthood, this was compared between PND 72–75 and PND 39 rats at each frequency. In the LAT, LFP facilitation was significantly greater in PND 39 rats compared to PND 72–75 rats at 20 Hz (Fig. 1B, middle; age x pulse interaction p = 0.0001, F(9,180) = 4.019, main effect of age p = 0.1946, F(1,20) = 1.801, two-way RM-ANOVA, n = 11 PND 72–75 rats, n = 11 PND 39 rats), but was similar at 10 Hz or 40 Hz (10 Hz: age x pulse interaction p = 0.9651, F(9,180) = 0.3274, main effect of age p = 0.6860, F(1,20) = 0.1683; 40 Hz: age x pulse interaction p = 0.4349, F(9,180) = 1.134, main effect of age p = 0.4349, F(1,20) = 0.6350, two-way RM-ANOVA).

In the BA, LFP facilitation was lower in PND 39 rats compared to PND 72–75 rats at 10 Hz and 20 Hz (Fig. 1D; 10 Hz: age x pulse interaction p = 0.2318, F(9,180) = 1.315, main effect of age p = 0.0270, F(1,20) = 5.692; 20 Hz: age by pulse interaction p = 0.0038, F(9,180) = 2.836, main effect of age p = 0.0251, F(1,20) = 5.864), with similar suppression at 40 Hz in PND 72–75 and PND 39 rats (age x pulse interaction p = 0.9516, F(9,180) = 0.3620, main effect of age p = 0.9091, F(1,20) = 0.01336).

To compare across both age and frequency, the summation ratio was quantified (ratio >1 indicates *facilitation*, ratio <1 indicates *depression*). Consistent with facilitation measures, summation ratio was greater in the PND 39 LAT at 20 Hz compared to PND 72–75 LAT (Fig. 1C; p < 0.05 post-poc Holm-Sidak’s multiple comparisons test), but lower in the PND 39 BA at 10 and 20 Hz compared to PND 72–75 (Fig. 1E; p < 0.05 post-poc Holm-Sidak’s multiple comparisons test). This opposite pattern of age differences across LAT and BA makes it unlikely that the same cellular changes account for age difference in these nuclei.

Age differences in the facilitation of LFPs can be due to many factors. Two prominent factors could be immaturity of PFC glutamatergic excitatory drive to BLA in adolescence or immaturity of GABAergic inhibitory circuits recruited by PFC inputs to BLA in adolescence. If the substantial suppression at 40 Hz reflects greater recruitment of GABAergic circuits, the inverse is that low frequencies (10 Hz) may be a better gauge of excitatory inputs. Mid-range frequencies (20 Hz) may reflect a balance between glutamatergic and GABAergic influences. The pattern observed in the LAT, of less suppression at 20 Hz, might hint toward weaker inhibition of LAT in adolescents that has not yet reached the mature adult level (with similar degrees of maximal inhibition when pushed at 40 Hz), while the pattern observed in the BA, of less facilitation at 10 Hz and 20 Hz, might hint toward immature excitatory drive of BA in adolescents. To begin to parse glutamatergic and GABAergic causes for age differences, the effects of PFC train stimulation was measured after local blockade of GABA_A_ receptors (PTX, 10 pmol/100 nL in ACSF) or vehicle (n = 20 hemispheres from 16 rats at each age, divided in a counterbalanced 2 × 2 design between (LAT or BA recordings) x (PTX or vehicle), Supplementary Figs 1 and 2; rats from the study above were examined here after local administration (n = 11 rats/age) in addition to another 5 rats/age). The expectation is that if age differences are caused by weaker GABAergic systems in adolescence, the age differences will be diminished by PTX. In contrast, if age differences are due to weaker excitatory drive in adolescence, the age differences will still exist after PTX.

**Figure 2:**
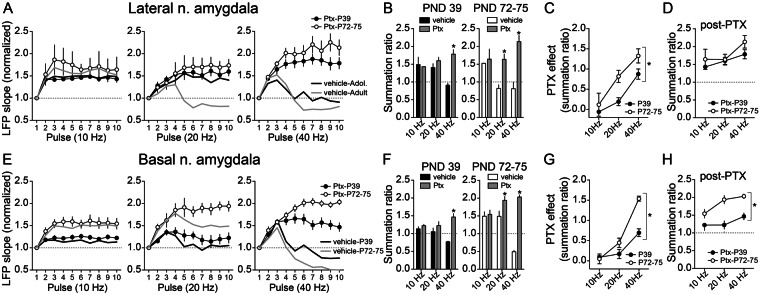
Age differences in sensitivity to blockade of GABA-A receptors. Picrotoxin (PTX) or vehicle was administered into the BLA while recordings LFPS. **(A)** In the LAT, PTX had minimal effect on facilitation/depression of LFPs at 10 Hz (p > 0.05, two-way ANOVA; compare vehicle plot with PTX plot), but it significantly increased facilitation of LFPs in PND 72–75 rats at 20 Hz (p < 0.05, two-way ANOVA), with minimal impact in PND 39 rats. PTX significantly reversed the depression of LFPs at 40 Hz in both PND 72–75 and PND 39 LAT (p < 0.05, two-way ANOVA both comparisons). **(B)** The same pattern was observed upon measurement of LAT summation ratio, where PTX reversed the depression at 40 Hz in PND 72–75 and PND 39 rats (*p < 0.05, post-hoc Holm-Sidak’s multiple comparisons test after two-way ANOVA; compare vehicle plot with PTX plot), and additionally at 20 Hz in PND 72–75 rats (*p < 0.05, post-hoc Holm-Sidak’s multiple comparisons test after two-way ANOVA). **(C)** The magnitude of the effect of PTX was measured across age in the LAT. PTX had significantly greater impact in PND 72–75 rats compared to PND 39 rats (p < 0.05, two-way ANOVA). **(D)** The LAT summation ratio after PTX was similar in PND 72–75 and PND 39 rats (p > 0.05, two-way ANOVA), suggesting that a difference in GABAergic function is sufficient to explain age differences in LAT summation of LFPs. **(E)** In the BA, PTX had minimal effect on summation of LFPs at 10 Hz (p > 0.05, two-way ANOVA), but it significantly increased LFP facilitation in adults at 20 Hz (p < 0.05, two-way ANOVA), with minimal impact in PND 39 rats. PTX significantly reversed the depression of LFPs at 40 Hz in both PND 72–75 and PND 39 BA (p < 0.05, two-way ANOVA both comparisons). This pattern was similar to effects in LAT. **(F)** Also similar to LAT, PTX in the BA reversed the suppression of summation at 40 Hz in PND 72–75 and PND 39 rats (*p < 0.05, post-hoc Holm-Sidak’s multiple comparisons test after two-way ANOVA), and additionally increased summation at 20 Hz in PND 72–75 rats (*p < 0.05, post-hoc Holm-Sidak’s multiple comparisons test after two-way ANOVA). **(G)** The magnitude of the effect of PTX in the BA was significantly greater in PND 72–75 compared to PND 39 rats (*p < 0.05, two-way ANOVA). **(H)** When compared after PTX there was still a significant difference in BA summation ratios between PND 72–75 and PND 39 rats (*p < 0.05, two-way ANOVA). This indicates that GABAergic inhibition is not likely to explain age differences in LFP summation in BA.

Consistent with low frequency reflecting primarily excitatory inputs, and not strongly reflecting GABAergic factors that suppress facilitation, we found that PTX did not significantly impact LAT LFP facilitation at 10 Hz in PND 72–75 rats (Supplementary Figs 3 and 4, Fig. 2A, left; n = 5 rats/group; drug x stimulus number p = 0.2721, F(9,72) = 1.263, main effect of drug p = 0.8998, F(1,8) = 0.01688, two-way RM-ANOVA) or PND 39 rats (Fig. 2A, left; n = 5 rats/group; drug x stimulus number p = 0.9471, F(9,72) = 0.3670, main effect of drug p = 0.1737, F(1,8) = 2.231, two-way RM-ANOVA). PTX boosted LAT LFPs at 40 Hz in PND 72–75 rats (Supplementary Figs 3 and 4, Fig. 2A, right; n = 5 rats/group; drug x stimulus number p < 0.0001, F(9,72) = 14.71, main effect of drug p = 0.0139, F(1,8) = 9.831, two-way RM-ANOVA) and PND 39 rats (n = 5 rats/group; drug x stimulus number p < 0.0001, F(9,72) = 13.60, main effect of drug p = 0.5484, F(1,8) = 0.3927, two-way RM-ANOVA), consistent with significant GABAergic modulation of LFP interaction at 40 Hz. However, PTX boosted LAT LFPs at 20 Hz only in PND 72–75 rats, but had weak effect in PND 39 rats (Supplementary Figs 3 and 4, Fig. 2A, middle; PND 72–75 n = 5 rats/group; drug x stimulus number p < 0.0001, F(9,72) = 25.60, main effect of drug p = 0.0278, F(1,8) = 7.195, two-way RM-ANOVA; PND 39 n = 5 rats/group; drug x stimulus number p = 0.0756, F(9,72) = 1.840, main effect of drug p = 0.1314, F(1,8) = 2.824, two-way RM-ANOVA). Because PTX has a robust effect at 20 Hz in adult rats but not in adolescent rats, one interpretation is that LAT GABAergic circuits are only weakly engaged at this intermediate frequency in adolescent rats. Indeed, consistent with this interpretation, the effect of PTX on summation ratio in LAT was greater in PND 72–75 compared to PND 39 rats (Fig. 2C; main effect of age p = 0.0044, F(1,25) = 9.78, two-way ANOVA) and LFPs were similar at PND 39 and PND 72–75 for all frequencies after GABA_A_ receptors were blocked with PTX, including 20 Hz, whether measuring facilitation across the train (Fig. 2B; frequency x age interaction p = 0.6316, F(2,25) = 0.4681, main effect of age p = 0.1478, F(1,25) = 2.231, two-way ANOVA) or summation ratio (Fig. 2D; main effect of age p = 0.148, F(1,25) = 2.231; age x frequency interaction p = 0.632, F(2,25) = 0.468, two-way ANOVA).

In the BA, there was no significant effect of PTX at low frequencies (10 Hz) in PND 72–75 or PND 39 rats (Supplementary Figs 3 and 4, Fig. 2E, left; PND 72–75 n = 5 rats/group; drug x stimulus number p = 0.9692, F(9,72) = 0.3104, main effect of drug p = 0.8290, F(1,8) = 0.04981, two-way RM-ANOVA; PND 39 n = 5 rats/group; drug x stimulus number p = 0.0946, F(9,72) = 1.744, main effect of drug p = 0.4778, F(1,8) = 0.5545, two-way RM-ANOVA) but PTX had effect at high frequencies (40 Hz) in PND 72–75 and PND 39 (Supplementary Figs 3 and 4, Fig. 2E, right; PND 72–75 n = 5 rats/group; drug x stimulus number p < 0.0001, F(9,72) = 101.5, main effect of drug p = 0.0019, F(1,8) = 20.46, two-way RM-ANOVA; PND 39 n = 5 rats/group; drug x stimulus number p < 0.0001, F(9,72) = 11.51, main effect of drug p = 0.3794, F(1,8) = 0.8655, two-way RM-ANOVA), similar to effects of PTX in LAT. There were intermediate effects of PTX at 20 Hz in PND 72–75 rats (Supplementary Figs 3 and 4, Fig. 2E, middle; n = 5 rats/group; drug x stimulus number p < 0.0001, F(9,72) = 5.762, main effect of drug p = 0.0616, F(1,8) = 4.719, two-way RM-ANOVA) but still no effect in PND 39 rats (n = 5 rats/group; drug x stimulus number p = 0.4252, F(9,72) = 1.030, main effect of drug p = 0.612, F(1,8) = 0.2960, two-way RM-ANOVA). The effect of PTX was different between PND 72–75 and PND 39 rats (Fig. 2G; main effect of age p = 0.0001, F(1,24) = 21.01, two-way ANOVA). Further, even after PTX, PND 72–75 and PND 39 BA LFPs differed from each other at all frequencies, when measuring across trains (Fig. 2F; frequency x age interaction p = 0.1410, F(2,25) = 2.128, main effect of age p < 0.0001, F(1,25) = 46.20, two-way ANOVA) or the summation ratio (Fig. 2H; main effect of age p < 0.0001, F(1,24) = 46.20; age x frequency interaction p = 0.141, F(2,24) = 2.128, two-way ANOVA). One interpretation is that, while GABAergic systems can influence BA facilitation, differences in GABAergic systems do not appear to account for age differences of BA LFP facilitation/depression.

### BLA neuron responses to PFC

**Figure 3:**
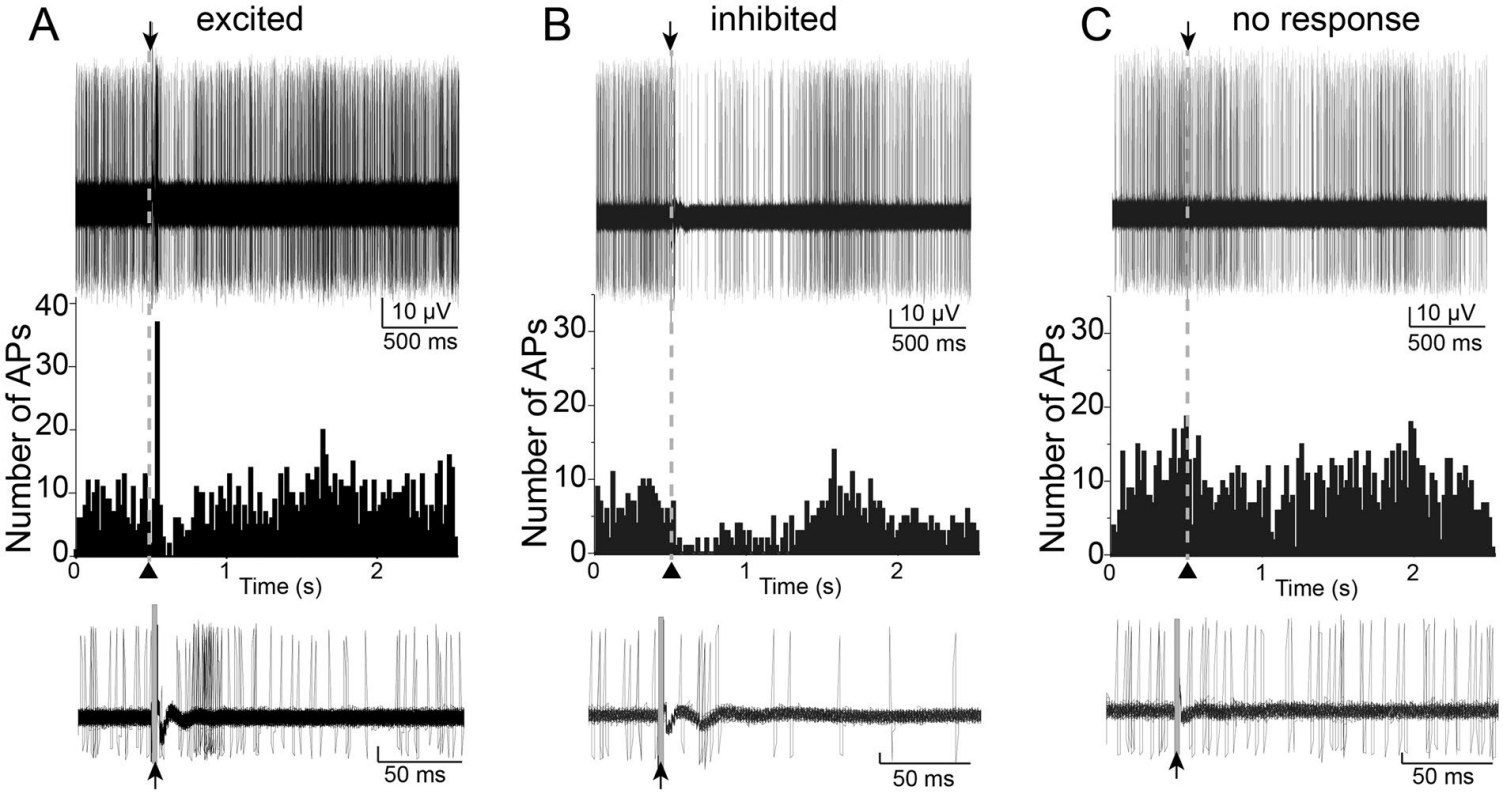
Patterns of neuronal response to PFC stimulation. Single neurons were recorded throughout the BLA. Three types of responses were characterized based on criteria (Methods). Shown in all the examples here are 50 consecutive overlaid traces of the response to stimulation (top), the peristimulus time histograms (middle) and overlaid traces of the responses at a smaller time scale (bottom). Stimulation occurred at the arrowheads. **(A)** Example of a neuron that displayed excitation in response to PFC stimulation. **(B)** Example of a neuron that displayed inhibition in response to PFC stimulation. **(C)** Example of a neuron that displayed no response to PFC stimulation. Stimulation artifacts were blanked for clarity.

**Figure 4:**
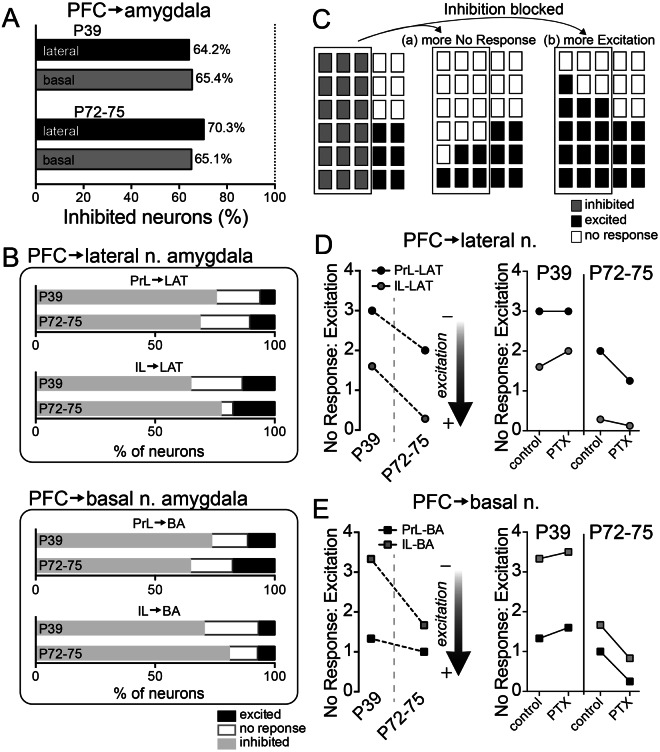
Proportion of response types across age. The response of BLA neurons to PFC stimulation was characterized by response type (Excitation, Inhibition, No Response) and by stimulation location (prelimbic (PrL) or infralimbic (IL) PFC). **(A)** The majority of BLA neurons in PND 72–75 and PND 39 rats had an Inhibition response type upon PrL or IL stimulation. **(B)** The distribution of Excitation and No Response measured in the LAT and BA demonstrate their occurrence (~30–35%) relative to all responses. **(C)** Parts-of-whole plot to illustrate the distribution of response types under baseline conditions (left), and two possible outcomes after blockade of inhibition with PTX. Each plot represents a population of BLA neurons, with each rectangle representing a neuron. The outlined neurons on the left represent BLA neurons that show a GABA_A_-mediated inhibitory response. When this inhibitory response is blocked by PTX these neurons might mostly respond to PFC in one of two ways: (a) the previously Inhibited neurons are now mostly No Response, or (b) the previously Inhibited neurons are now mostly Excited. **(D)** The ratio of No Response to Excitation types in LAT was quantified to gain insight into their relative distribution across age. There was a shift towards lower values (more Excitation) in PND 72–75 rats. PTX shifted values towards more Excitation in PND 72–75 rats (IL → LAT, PrL → LAT, p < 0.05, χ^2^), but more No Response in PND 39 rats (IL → LAT, PrL → LAT, p < 0.05, χ^2^). **(F)** The ratio of No Response to Excitation types in BA was quantified. There was a shift towards lower values (more Excitation) in PND 72–75 rats. PTX shifted values towards more Excitation in PND 72–75 rats (IL → BA, PrL → BA, p < 0.05, χ^2^), but more No Response in PND 39 rats (IL → BA, PrL → BA, p < 0.05, χ^2^).

The results from LFP experiments demonstrate similar degrees of maximal BLA inhibition in adults and adolescents recruited by high PFC stimulation (40 Hz), but clear age differences in facilitation/depression at intermediate levels of PFC stimulation (20 Hz). Furthermore, the age differences at this intermediate level were opposite between LAT and BA. This points toward different mechanisms in the LAT and BA that underlie age differences: less inhibition over LAT in adolescence but weaker PFC excitatory drive to BA in adolescence. LFP facilitation/depression indirectly indicates a capacity for inhibition of BLA neuron activity. While this capacity is highly important, it is ultimately the firing of BLA neurons that will determine BLA output. To more directly test age differences in PFC influences on BLA, the response of single BLA neurons upon PFC stimulation was measured before and after vehicle or PTX administration (using the rats from above (n = 20 rats/age) and additional rats (additional n = 29 hemispheres from 16 P72–75 rats for a total of 49 hemispheres from 36 adult rats: control n = 27 hemispheres, vehicle n = 11 hemispheres, PTX n = 11 hemispheres; additional n = 33 hemispheres from 18 P39 rats for a total of 53 hemispheres from 38 adolescent rats: control n = 29 hemispheres, vehicle n = 12 hemispheres, PTX n = 12 hemispheres); Control recordings were performed with no local administration; Supplementary Fig. 5). These same rats were used to obtain the data for all subsequent results. The response of BLA principal neurons to PFC stimulation was categorized as Excitatory, Inhibitory, or No response (Fig. 3A–C; Materials and Methods). Neurons categorized as Inhibited displayed a suppression of spontaneous firing that began within a short-latency (<30 ms) after PFC stimulations (Fig. 3B). More often than not, PFC stimulation evoked an inhibitory effect on neurons of the LA (Fig. 4A; 64/91 neurons, p < 0.0001, two-tailed binomial test) and BA (56/86 neurons, p = 0.003, two-tailed binomial test) in PND 72–75 rats. In PND 39 rats, similar results were obtained (Fig. 4A; LA: 61/95 neurons, p = 0.0037, two-tailed binomial test; BA: 70/107 neurons, p = 0.0009, two-tailed binomial test). To refine results, subdivisions of PFC were examined more closely.

The distribution of response types between PND 72–75 and PND 39 (Fig. 4B; Inhibitory, Excitatory, and No response) was similar for all input combinations: PrL → BA (χ^2^ = 0.918, df = 3, p = 0.821; PND 72–75 = 39 neurons, PND 39 = 61 neurons); PrL → LAT (χ^2^ = 0.771, df = 3, p = 0.856; PND 72–75 = 53 neurons, PND 39 = 54 neurons); IL → BA (χ^2^ = 1.760, df = 3, p = 0.624; PND 72–75 = 45 neurons, PND 39 = 46 neurons); IL → LAT (χ^2^ = 4.715, df = 3, p = 0.194; PND 72–75 = 47 neurons, PND 39 = 43 neurons). All these analyses indicate a heavily Inhibitory response irrespective of age. While the heavily Inhibitory response profile may be similar between age groups, the underlying causes for an abundance of Inhibitory types may be different. Indeed, the LFP data suggest that there may be different causes for a skew towards Inhibitory outcomes across age, with weaker PFC excitatory drive in adolescents but greater PFC inhibitory effects in adults. Similarly, a skew towards an Inhibitory response phenotype may be caused by either (a) weaker PFC excitatory drive to BLA projection neurons so that Inhibitory response types are favored over Excitatory response types or (b) greater recruitment of inhibitory circuits. Put operationally, in the balance between response types, Inhibitory is more prominent than Excitatory because (a) Excitatory responses are subthreshold or absent and measured as Non-Responders or (b) Excitatory responses are inhibited and measured as Inhibitory.

If PFC inputs are weaker in one group, it is expected that a higher proportion of neurons would be No response due to a weaker, subthreshold impact on firing. To examine this further, the proportion of Non-responding neurons relative to Excited neurons was examined. The ratio was higher in PND 39 rats (Fig. 4D,E, left; main effect of age p = 0.0318, F(1,3) = 14.50, two-way ANOVA). This may hint towards overall weaker PFC inputs to the BLA in PND 39 rats.

While this is suggestive of a mechanistic difference between adults and adolescents, the Inhibitory response may mask accurate comparison of Excitatory and Non-responses. Therefore, this ratio was assessed again upon local intra-BLA blockade of GABA_A_ receptors with PTX via cannula infusion compared to vehicle. This blocks the Inhibitory phenotype, but the neurons that normally show an Inhibitory phenotype may still respond to PFC in a different manner (Excitatory) or now show No response. After blockade of the Inhibitory phenotype, a shift in the relative distribution of response types towards No response would indicate that the dearth of Excitatory phenotypes is due to weak excitation of projection neurons (a, above; Fig. 4C). In contrast, a shift toward Excitatory responses would indicate that inhibition actively suppressed excitation and decreased the incidence of Excitatory response types (b, above; Fig. 4C). Overall, PTX caused opposite changes in PND 72–75 and PND 39 rats (comparison of No Response:Excitation ratio, Age x PTX interaction, p = 0.0033, F(1,6) = 22.11, two-way RM-ANOVA), with a small shift towards No Response in adolescents and a shift towards Excitation in PND 72–75.

We next tested whether this shift from adolescence to adults was observable across all PFC → BLA inputs. In PND 72–75 rats, PTX caused a significant shift toward Excitatory responses in LAT (Fig. 4D, right; PrL: χ^2^ = 4.04, df = 1, p = 0.045, control = 5/48 neurons, PTX = 4/9 neurons; IL: χ^2^ = 7.20, df = 1, p = 0.0073, control = 7/40 neurons, PTX = 8/9 neurons) and in BA (Fig. 4E right; PrL: χ^2^ = 6.94, df = 1, p = 0.0085, control = 6/34 neurons, PTX = 8/10 neurons; IL: χ^2^ = 8.15, df = 1, p = 0.0043, control = 3/42 neurons, PTX = 7/11 neurons). Thus, the blockade of GABA_A_-mediated inhibition uncovers additional excitation in PND 72–75 rats. However, in PND 39 rats, PTX shifted the response profile in LAT towards No response upon PFC stimulation (Fig. 4D, right; PrL: χ^2^ = 5.23, df = 1, p = 0.022, control = 9/49 neurons, PTX = 8/12 neurons; IL: χ^2^ = 4.87, df = 1, p = 0.027, control = 8/43 No response neurons, PTX = 8/12 neurons) and in BA (Fig. 4E, right; PrL: χ^2^ = 6.21, df = 1, p = 0.013, control = 8/53 neurons, PTX = 8/13 neurons; IL: χ^2^ = 4.27, df = 2, p = 0.039, control = 10/44 No response neurons, PTX = 7/9 neurons). Thus, blockade of GABA_A_-mediated inhibition in PND 39 rats does not uncover significant excitation. These data indicate that the relatively low incidence of Excitatory responses to PFC stimulation in PND 72–75 rats is partly due to GABAergic inhibition, but the low incidence of Excitatory responses in adolescents is partly due to weaker excitatory effects of PFC inputs to LAT and BA.

### Limited PFC regulation over adolescent amygdala

**Figure 5:**
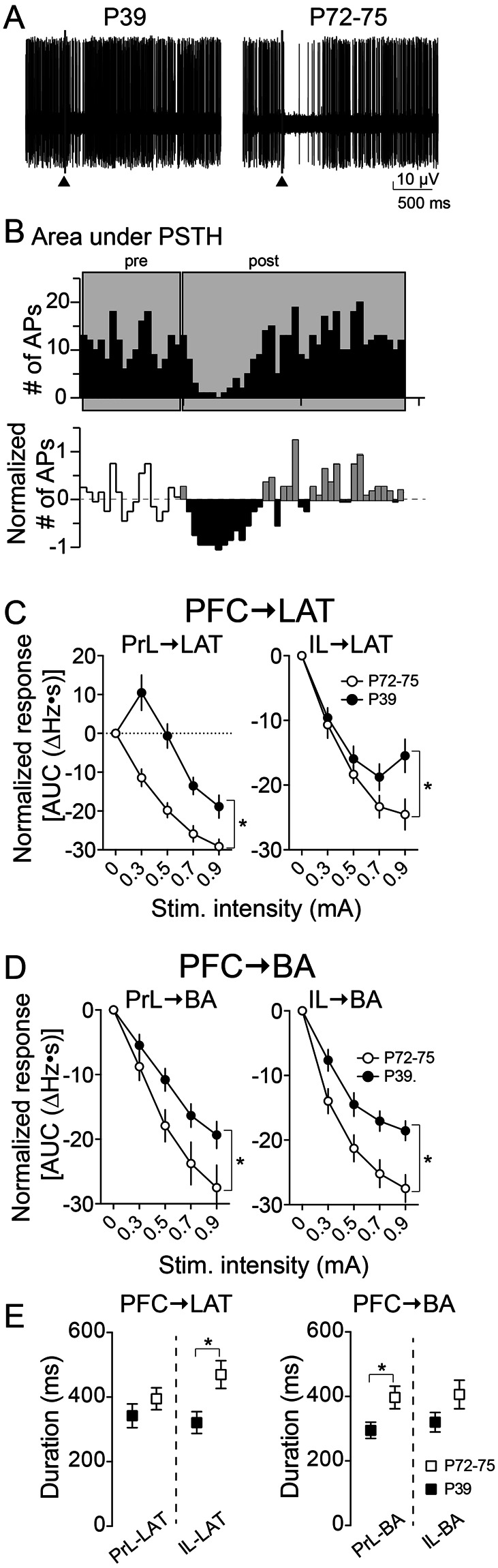
Age differences in integrated PFC-evoked inhibition of BLA neuron firing. A portion of BLA neurons were categorized as Inhibited by single pulse stimulation of PFC in PND 72–75 and PND 39 rats. **(A)** Shown here is an example of a BLA neuron from a PND 39 rat (left) and PND 72–75 rat (right) that fit criteria for Inhibition response type. Fifty consecutive overlaid traces are shown here of the response to the same stimulation intensity. Stimulation occurs at the arrowhead. Stimulation artifacts were blanked for clarity. **(B)** The response to PFC stimulation was quantified from Inhibited neurons as the Area Under the peristimulus time histogram Curve (AUC). **(C)** There was weaker inhibition evoked by PFC stimulation in the LAT of PND 39 rats compared to PND 72–75 rats (PrL → LAT, IL → LAT, *p < 0.05, two-way ANOVA for both comparisons). **(D)** Similarly, in the BA there was significantly less inhibition in PND 39 PrL → BA and IL → BA compared to PND 72–75 (*p < 0.05, two-way ANOVA for both comparisons). **(E)** The duration of inhibition was quantified and found to be significantly greater in PND 72–75 rats compared to PND 39 rats for IL → LAT responses (*p < 0.05, post-hoc Holm-Sidak’s multiple comparisons test after two-way ANOVA) and for PrL → BA (*p < 0.05, post-hoc Holm-Sidak’s multiple comparisons test after two-way ANOVA).

The neuronal response profile is a rough indication of the overall influence of an input. This can yield important clues about a shift in excitation and inhibition across age. But this measure does not capture the magnitude of the inhibitory response, nor its temporal aspects. To determine if the strength of inhibition varies across age, inhibition evoked by PFC stimulation (Fig. 5A) was quantified as the area under PSTHs (Inhibition_AUC_; Fig. 5B). Only Inhibited neurons with a full input-output curve were included in this analysis.

Inhibition_AUC_ was dependent upon the stimulation intensity (Fig. 5C,D) and was weaker in PND 39 rats (Fig. 5A,C,D). Inhibition_AUC_ was different across age at all PFC inputs to the BLA (Fig. 5C,D; age x stimulation interactions, PrL → LAT p < 0.0001, F(4,44) = 10.85, n = 7 neurons PND 39, n = 6 neurons PND 72–75; IL → LAT p = 0.0128, F(4,48) = 3.553, n = 8 neurons P39, n = 6 neurons PND 72–75; PrL → BA p = 0.0053, F(4,48) = 4.211, n = 7 neurons PND 39, n = 7 neurons PND 72–75; IL → BA p = 0.0002, F(4,48) = 6.661, n = 7 neurons PND 39, n = 8 neurons PND 72–75, from 10 rats PND 39 and 11 rats PND72–75, two-way RM-ANOVAs), with a lower Inhibition_AUC_ in PND 39 rats across all inputs, consistent with weaker inhibitory effects of PFC inputs to BLA in PND 39 rats. The duration of inhibition was significantly shorter in PND 39 compared to PND 72–75 rats (Fig. 5E, left; main effect of age, p = 0.0124, F(1,23) = 7.354, two-way ANOVA).

### Maturation of Excitatory inputs

**Figure 6:**
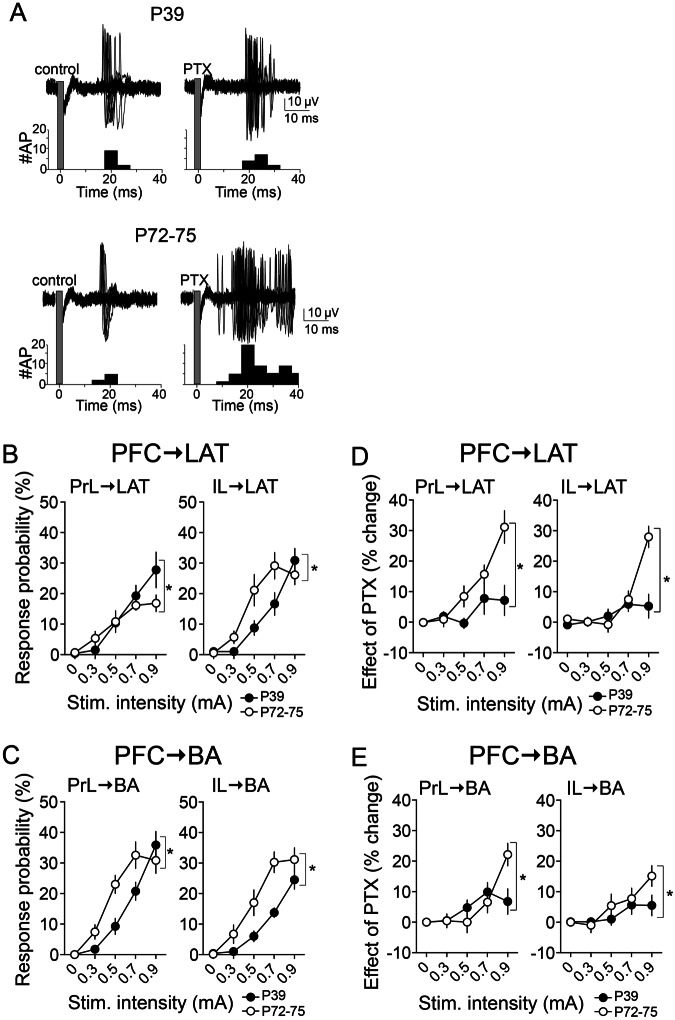
Age differences in excitation of BLA neurons caused by PFC inputs. (**A)** BLA neurons that fit criteria for Excitatory responses were tested. These neurons displayed apparent monosynaptic responses to PFC stimulation (see Methods), and these responses were enhanced by blockade of GABA_A_ receptors by intra-BLA PTX. Shown here are overlaid traces above peristimulus time histograms. Stimulation occurs at the grey bar. Stimulation artifacts were blanked for clarity. **(B)** The excitatory response of LAT neurons to IL inputs was lower in PND 39 rats (*p < 0.05, two-way ANOVA). **(C)** While the maximal response of BA neurons was similar across groups, overall the response to PrL and IL inputs was lower in PND 39 rats. **(D)** Intra-BLA PTX exerted significantly greater effects on PrL → LAT and IL → LAT responses recorded from PND 72–75 compared to PND 39 rats (*p < 0.05, two-way RM-ANOVA for both plots). **(E)** Similarly, Intra-BLA PTX exerted significantly greater effects on PrL → BA and IL → BA responses recorded from PND 72–75 compared to PND 39 rats (*p < 0.05, two-way RM-ANOVA for both plots).

The results above demonstrate a shift in the effects of PFC inputs to BLA between adolescence and adulthood, and that this can be accounted for by immaturity of GABAergic influences in LAT with a potential additional component of immaturity of glutamatergic drive in BA. The contribution of the glutamatergic element can be tested directly by measurement of the BLA neuron excitatory response to PFC stimulation. The probability of a monosynaptic response to stimulation was quantified across PFC inputs to BLA principal neurons that showed an Excitatory response profile (Fig. 6A), with the hypothesis that immature innervation of PFC → BLA would produce weaker response in adolescence.

The LAT response to inputs from PFC was only weaker in PND 39 rats in the IL → LAT input (Fig. 6B, right; age x stimulation interaction, p = 0.0029, F(4,52) = 4.626; main effect of age, p = 0.1086, F(1,13) = 3.169, two-way RM-ANOVA, n = 7 neurons PND 72–75, n = 8 neurons PND 39), while the excitatory impact of PrL → LAT was marginally stronger in PND 39 rats compared to PND 72–75 (Fig. 6B, left; age x stimulation interaction, p = 0.0426, F(4,52) = 2.664; main effect of age, p = 0.4338, F(1,13) = 0.6523, two-way RM-ANOVA, n = 7 neurons PND 72–75, n = 8 neurons PND 39). In contrast, all inputs to BA were weaker in adolescents, PrL → BA (Fig. 6C, left; age x stimulation interaction, p = 0.0025, F(4,52) = 4.717; main effect of age, p = 0.0606, F(1,13) = 4.223, two-way RM-ANOVA, n = 7 neurons PND 72–75, n = 8 neurons PND 39) and IL → BA (Fig. 6C, right; age x stimulation interaction, p = 0.0014, F(4,52) = 5.164; main effect of age, p = 0.0080, F(1,13) = 9.766, two-way RM-ANOVA, n = 7 neurons PND 72–75, n = 8 neurons PND 39). These results indicate age differences in the excitatory drive of PFC inputs to BLA. However, these results are likely under GABAergic influence, possibly interfering with the conclusions about age differences in the excitatory effects of PFC inputs.

To test the excitatory drive in the absence of GABA_A_ influences, experiments were performed after local intra-BLA infusion of PTX or vehicle (as above), and excitatory drive was measured (n = 5 rats/group). When GABA_A_-mediated inhibition was blocked, IL → LAT inputs remained weaker in PND 39 confirming weaker excitatory strength of this input (age x stimulation interaction, p = 0.0001, F(4,56) = 6.984; main effect of age, p = 0.0011, F(1,14) = 16.60, two-way RM-ANOVA, n = 8 neurons PND 72–75, n = 8 neurons PND 39), and PrL → LAT inputs were no longer different between PND 72–75 and PND 39 rats confirming similar excitatory strength of this input (age x stimulation interaction, p = 0.1745, F(4,52) = 1.655; main effect of age, p = 0.0689, F(1,13) = 3.931, two-way RM-ANOVA, n = 7 neurons PND 72–75, n = 8 neurons PND 39). When GABA_A_-mediated inhibition was blocked and BA neuronal responses were measured, both PFC → BA inputs remained weaker in adolescents confirming weaker excitatory strength of these inputs [PrL → BA (age x stimulation interaction, p = 0.1517, F(4,52) = 1.757; main effect of age, p = 0.0277, F(1,13) = 6.142, two-way RM-ANOVA, n = 7 neurons PND 72–75, n = 8 neurons PND 39) and IL → BA (age x stimulation interaction, p = 0.0001, F(4,52) = 7.219; main effect of age, p = 0.0004, F(1,13) = 21.93, two-way RM-ANOVA, n = 7 neurons PND 72–75, n = 8 neurons PND 39)]. This supports the conclusion that there is weaker excitatory impact of PFC inputs to adolescent BLA *in vivo*.

Despite weaker overall excitatory strength of PFC → BLA in adolescents, the measured responses to PFC before blockade of GABA_A_ influences were not dramatically different across age at the highest stimulation intensity. This could be caused by heavier GABA_A_ influences over the PFC → BLA response in adults that countermand the increased excitatory strength of PFC → BLA. To test if there is greater GABA_A_-mediated regulation PFC → BLA in adults, the effect of PTX on PFC → BLA excitatory responses was compared across groups (PTX – ACSF mean) in the same rats examined above. Indeed, there was a greater impact of PTX on PFC → BLA excitatory responses in PND 72–75 rats for PFC → LAT (Fig. 6D; PrL → LAT age x stimulation interaction, p = 0.0003, F(4,52) = 6.334; main effect of age, p = 0.0223, F(1,13) = 6.726, two-way RM-ANOVA, n = 7 neurons PND 72–75, n = 8 neurons PND 39; IL → LAT age x stimulation interaction, p < 0.0001, F(4,56) = 16.28; main effect of age, p = 0.0656, F(1,14) = 3.990, two-way RM-ANOVA, n = 8 neurons PND 72–75, n = 8 neurons PND 39) and PFC → BA (Fig. 6E; PrL → BA age x stimulation interaction, p = 0.0002, F(4,52) = 6.696; main effect of age, p = 0.5999, F(1,13) = 0.2891, two-way RM-ANOVA, n = 7 neurons PND 72–75, n = 8 neurons PND 39; IL → BA age x stimulation interaction, p = 0.1190, F(4,52) = 1.931; main effect of age, p = 0.2231, F(1,13) = 1.637, two-way RM-ANOVA, n = 7 neurons PND 72–75, n = 8 neurons PND 39). This supports the conclusion that GABAergic regulation is a major component that dictates the stronger impact of PFC → BLA in adults. However, even in the absence of this regulation, PFC → BLA inputs exert a greater impact in adults.

## Discussion

The BLA response to extrinsic inputs includes a heavily inhibitory component^32,54–56^. The current findings demonstrate a common theme, whether measured by evoked LFPs or single neuron responses, that PFC inputs to BLA do not exert as potent a response in adolescents compared to adults. While evidence emerges for a weaker inhibitory regulation in LAT and BA during adolescence, the primary contributing mechanism for this weakness is region specific. In the LAT there is weaker evoked inhibition in adolescents, whereas in the BA there is a weaker excitatory influence of PFC inputs. This is demonstrated by differences in the summation of LFPs, their sensitivity to PTX, and differences in the proportion of response types. The resultant inhibitory regulation of PFC over LAT and BA is weaker in adolescents, demonstrated by less PFC-evoked inhibition of neuronal firing. This is consistent with recent findings that the potency of PFC inputs to BLA changes across development^31^ and *in vivo* and *in vitro* studies that indicate weaker GABAergic regulation in prepubertal rats^57,58^.

There are some limitations of the current findings. While it is important to examine these circuits *in vivo*, anesthesia limits the generalizability of these findings. In addition, PFC was activated by electrical stimulation that can have some spatial spread. The stimulation intensities were kept below 1.0 mA to limit current spread but there may be a small degree of overlap between effects of PrL and IL stimulation. However, we do not believe that this overlap is near to complete because experiments where both PrL and IL were stimulated in series using two aligned stimulation probes still produced different effects. Additional confounds can arise, such as recruitment of multisynaptic paths to the BLA during train stimulation or antidromic activation of BLA circuits. While these potential confounds cannot be ignored, the overall conclusions would still point to less regulation of the adolescent BLA.

The relationships between maturation of the excitatory and inhibitory influences of PFC inputs to BLA may be inter-related. PFC → BLA can evoke EPSPs in BLA projection neurons that drive action potential firing^34,44,59^. Weaker PFC → BLA may produce smaller EPSPs and produce lower probability of action potential firing. PFC → BLA also excites BLA interneurons^31,32,34,44^ that exert an inhibitory effect on BLA projection neurons that is rapid enough to curtail the direct PFC → BLA excitatory effects on projection neurons^34,44^. Weaker PFC → BLA interneurons therefore may also produce less inhibition. This co-occurring shift in excitatory and inhibitory effects produced by weaker PFC inputs may account for a similar distribution of Excitatory/Inhibitory response profiles across age.

The nature of the frequency dependence of the PFC → BLA supports a mechanism that includes a shift in the balance between excitatory and inhibitory inputs. Single pulse stimulation of PFC evoked a rapid inhibition that peaked 40–50 ms after onset, and then decayed. Our data from single units and after PTX suggests that this occurs overlayed on a background of weaker excitatory inputs with a rapid onset and offset within a 10 ms window. When a second stimulation occurs at an interval shorter than 40–50 ms (before the inhibition caused by the first stimulation has peaked), one might expect that the inhibition caused by the first will have minimal impact on the response to the second stimulation, but the inhibitory component of the second stimulation can begin to overlap with the decay of the inhibitory component of the first stimulation. Stimulation trains at higher frequencies (e.g. with intervals of 10–25 ms, 100–40 Hz) would allow subsequent inhibitory events to add onto the decay of previous inhibitory events, with a result of increased inhibition across the train. Stimulation trains at lower frequencies (e.g. with intervals 50–100 ms, 20–10 Hz) would result in subsequent inhibitory events occurring during later phases of decay of the previous event, and less inhibitory impact. This fits well with the sensitivity of high frequency LFPs to PTX, and less sensitivity at lower frequencies, as well as the initial facilitation of LFPs observed during the first few pulses of 40 Hz trains. This concept has been documented in other regions, including cortex and hippocampus, where higher frequency stimulation (40 Hz) recruits substantial GABAergic inhibition^60^, and high frequency rhythms (gamma) are linked to the firing of fast-spiking interneurons^61–63^. The architecture of the BLA produces a similar outcome. There are different types of BLA GABAergic interneurons defined chemically, for instance cholecystokinin-containing (CCK) and parvalbumin-containing (PV) interneurons^64–66^, or electrophysiologically, for instance stuttering and fast-firing interneurons^67,68^. CCK interneurons have a maximal firing rate of ~20–30 Hz, while PV interneurons have a maximal firing rate >40 Hz^64,65^. BLA gamma frequency rhythmicity requires BLA PV fast-firing interneurons^69^. The importance of PV interneurons in gamma frequency oscillation is further guaranteed due to difference in short-term synaptic dynamics and frequency-dependence (between 10–40 Hz) of PV, CCK and axo-axonic interneurons^51,67^, with all types of BLA interneurons showing little frequency dependence of effects on BLA projection neurons at 1 Hz, and a near maximal effect when PV interneuron firing approaches 40 Hz^51,52^. Based on this, PV interneurons are expected to be increasingly recruited by excitatory inputs across a wide range of frequencies.

PFC excitatory influences may guide appropriate BLA-mediated behaviors while inhibitory influences may impose regulation over inappropriate BLA-mediated behaviors or control the magnitude of responses. The slow maturation of excitatory influences and resultant smaller afferent evoked inhibition^31^ is expected to produce a two-fold effect, weaker guidance of BLA-mediated behaviors and weaker regulation of these behaviors. This may produce age differences in behaviors that are sensitive to the balance between glutamate and GABAergic in the BLA, including generalization of conditioned fear and fear extinction^70–85^. Indeed, measures of reduced GABAergic influences, or a shift in the inhibitory-excitatory effects of PFC inputs to favor excitation have been observed after fear conditioning^30^, with opposite changes after extinction, such as decreased summation of LFPs^86^, and reduced excitatory impact of PFC inputs^44^. These behaviors are also linked to rhythmic activity in the BLA. Different types of BLA activity become dominant during specific BLA-mediated behaviors, and are an important component of BLA functions. BLA interneurons are recruited to theta synchrony in the presence of noxious stimuli^87^, BLA theta synchronization with sensory cortical regions occurs in conditions that require a BLA-mediated response during fear recall BLA^88^, and PFC-BLA synchrony at theta is observed during fear recall and expression^89,90^. Our results would indicate that theta frequency of inputs is expected to exert less inhibition over BLA, permitting greater BLA-mediated responses. In contrast, PFC-BLA (specifically IL-LAT) phase coherence shifts more towards IL leading^90^ and BLA fast and slow gamma (40–70 Hz) is coupled to PFC rhythms during conditions in which BLA-mediated behavioral responses should be suppressed, such as during safety cues^91^.

Top-down regulation of emotion matures across juvenile and adolescent years^17,92–94^. In parallel, there is structural maturation of PFC that lags behind amygdala maturation^11^, maturation of the connections and functional interactions between corticolimbic structures^12,92^, development of a reciprocal relationship between the activity of the PFC and amygdala^13,95,96^, and a shift in the PFC regions that exert the strongest influence over amygdala^16,97^.

PFC input density in the BLA reaches adult levels by early adolescence^38^, as does the amount of PFC neurons that project to the BLA^98^. The spine number^99^ and synaptic protein synaptophysin is stable throughout the adolescent to adult period^100^, consistent with maturity of the structural components of PFC excitatory inputs during adolescence. However, the response to excitatory synaptic inputs or synaptic function may continue to mature throughout adolescence. Adult patterns of BLA interneuron chemical phenotype appears by P30^101,102^, and basic GABAergic synaptic properties are mature by this same time^103^. However, there is substantial evidence that GABAergic systems in the BLA are still not fully matured until later ages. For instance, perineuronal nets (PNNs) in the BLA continue to mature throughout adolescence^104^. Immature elaboration of PNN is considered a hallmark of juvenile plasticity in cortex and related to GABAergic regulation that has not reached maturity^105,106^. This slower maturation is accompanied by increased GABA synthesis between 1–2 months postnatal^107^, and increased intra-BLA inhibition evoked by excitatory inputs to the BLA between juvenile and adult ages^57^, and a shift in the functional impact of GABAergic systems *in vivo*^58^. Adolescent state of these BLA GABAergic systems may contribute to the weaker GABAergic regulation of the adolescent BLA in the current study.

Disruptions in the normal maturation of PFC-amygdala functional connectivity can be caused by early life stress^20,108–112^, and is associated with anxiety, depression, autism and drug abuse^20,113–116^. The current findings refine and develop potentially vulnerable nodes of this circuitry that may produce psychiatric symptoms when disrupted.

## Methods

All experiments were approved by the Institutional Animal Care and Use Committee of Rosalind Franklin University, and experiments were carried out in accordance with the Guide for the Care and Use of Laboratory Animals (National Research Council, 2011). Care was taken to reduce any unnecessary distress to the animals and to limit the total number of animals used.

### Subjects

Male Sprague–Dawley rats (Harlan, Indianapolis, IN) were used in this study. They were housed 2–3 per cage in the Rosalind Franklin University animal facility with free access to food and water, and maintained on a reversed 12 h light/dark cycle (light cycle from 7:00 pm to 7:00 am). Adolescence can be defined by a range of behavioral features, and can encompass a wide range of ages^117^, from PND 28 to sexual maturity. The focus here is on rats that have not yet undergone puberty (approximately PND 42 in male rats). Prepubescent rats arrived at the animal facility on postnatal day (PND) 25 and electrophysiological recording were performed on PND 39. Adult rats arrived on PND 58 and electrophysiological recordings were performed on PND 72–75.

### *In vivo* extracellular recording

*In vivo* extracellular recordings were performed in anesthetized rats (urethane, Sigma-Aldrich, St. Louis, MO; 1.5 g/kg dissolved in 0.9% saline, intraperitoneally) as described^118^. Rats were placed in a stereotaxic device (Stoelting, Wood Dale, IL) after deep anesthesia was confirmed. Their body temperature was monitored via a rectal temperature probe, and maintained at 36–37 °C using a heating pad with a temperature controller (Model TC–1000, CWE Inc, Ardmore, PA). The BLA and PFC were localized using a stereotaxic atlas^119^. For adult rats, the coordinates used for the BLA centered on 4.8 mm–5.2 mm lateral, 2.5 mm–3.8 mm caudal from bregma. The coordinates used for PFC were 0.7 mm lateral, 2.7 anterior from bregma, and 3.9 ventral (PrL) or 5.1 ventral (IL) from the brain surface. For adolescent rats, coordinates were scaled according to the measured distance between the bregma and interaural skull landmarks. Burr holes were drilled in the skull at locations overlying the BLA and the PFC. A bipolar concentric stimulation electrode (Rhodes Medical Instruments CA, USA or MicroProbes, Gaithersburg, MD, USA) was lowered into the IL or PrL. Single–barrel recording electrodes were constructed from glass pipettes (2.0 mm outer diameter, World Precision Instruments, Sarasota, FL), pulled using a vertical microelectrode puller (PE–2; Narishige, Tokyo, Japan), and broken under microscopic view to produce a 1–2 µm diameter tip. The recording electrode was filled with 2% Pontamine Sky Blue (Alfa Aesar, Ward Hill, MA) in 2 M NaCl (Fisher Scientific, Pittsburgh, PA) and then slowly lowered into the amygdala via a hydraulic microdrive (Model MO–10, Narishige).

During extracellular recording, signals were amplified with a headstage connected to a preamplifier (2400 Amplifier, Dagan Corporation, Minneapolis, MN or 1800 Amplifier, A-M Systems, Sequim, WA), filtered at 0.1–0.3 Hz (low cut–off frequency) and 3 kHz (high cut–off frequency), and outputted simultaneously to an oscilloscope (Model 2532 BK Precision, Yorba Linda, CA) and an audio monitor (Model AM8, Grass Instruments, West Warwick, RI). In addition, amplified outputs were digitized (10 kHz; Model ITC–18, HEKA, Bellmore, NY) and fed to a personal computer (Mac Pro/2.8 Apple, Cupertino, CA), monitored using Axograph X software (Sydney, Australia) and stored on a hard disk for off-line analysis. The anesthesia state of the animal was monitored by cortical local field potential oscillations recorded from the concentric electrode in the PFC throughout recordings of neuronal firing. Animals were considered under deep anesthesia when the cortical field oscillations displayed a predominant slow (~1 Hz) rhythmic waveform.

#### PFC stimulation

Electrical stimulation was delivered to the IL or PrL through the implanted concentric bipolar electrode (Grass S88, Grass Instruments). Stimulation consisted of single pulses (0.2 Hz, 0.1–0.9 mA, 0.2 ms duration) or trains (10–40 Hz, 10 pulses, 0.1–0.9 mA, 0.1–0.2 ms duration).

#### Local field potential recordings

The approach for recording LFPs was modeled after approaches used to probe *in vivo* GABAergic regulation in cortical recordings with much success^60^. PFC was stimulated (as above; 0.1–0.9 mA) while the evoked local field potential was recorded. A stimulation intensity and duration was selected that evoked a field potential of approximately 50% the maximal amplitude. The PFC was stimulated at 10, 20, and 40 Hz (10 pulses/train, 10 s inter-stimulus interval). The initial slope of each evoked local field potential in the stimulus train was measured. In a set of experiments, the recording electrode was filled with recording solution and drug/vehicle for local application during recordings, as described below.

#### Single unit recordings

Single neurons were recorded throughout the BLA. Upon isolation of a single unit, baseline firing rates in spontaneously firing BLA projection neurons were recorded for 5 min before the PFC was electrically stimulated (as above, 0.1–0.9 mA, single pulses). Null stimulation (0 mA) data in the same pattern was obtained for comparison. A minimum of 40 sweeps at each stimulation intensity was acquired. The response of BLA neurons to PFC stimulation was measured as changes in the number of action potentials in peri-stimulus time analysis (see Data Analysis). In a set of experiments, drug/vehicle was delivered locally by cannula, as described below.

#### Microiontophoretic application of glutamate

Neurons of the BLA fire slowly under anesthesia, presenting difficulty for assessing inhibition of firing. Therefore, upon identification of a BLA neuron with an inhibitory response to PFC stimulation, slowly firing BLA neurons were induced to fire at 4–8 Hz with microiontophoretic application of glutamate. Multibarrel microelectrodes (4 barrels; A–M Systems) were constructed using a vertical microelectrode puller (PE–2; Narishige), and the tip was broken back under microscopic guidance. One barrel of the microelectrode was filled with 2% Pontamine Sky Blue (Alfa Aesar) in 2 M NaCl (Fisher Scientific) for electrophysiological recordings and a second barrel was filled with 1 M NaCl for automatic current balancing. One of the remaining barrels was filled with 50 mM glutamate (pH 8.0; Alfa Aesar) dissolved in 20 mM NaCl solution. The last barrel was empty. Glutamate was ejected with anodal iontophoretic current (E104B; Fintronics, Orange, CT). Retaining currents of the opposite polarity were used (10 nA) before and after ejection. The glutamate ejection current was adjusted to maintain a stable firing rate of BLA projection neurons. After a stable firing rate was achieved with glutamate iontophoresis, PFC was stimulated as above.

#### Local application of drugs

In specified experiments, intra-BLA local drug infusions were performed during electrophysiological recordings by one of two means^118^: (1) a cannula for infusions (pulled glass pipette with shank diameter 50–100 µm) was lowered into the BLA (15 degree angle off the rostral-caudal axis) and drugs were applied through this cannula while a different higher impedence electrode was used to measure the firing of BLA neurons; or (2) a low impedence single barrel recording electrode (>50 µm tip) was lowered into the BLA and LFPs were recorded with this electrode before and after drugs were applied through this electrode. Intra-BLA infusions of either artificial cerebrospinal fluid vehicle (ACSF, prepared as below) or picrotoxin (PTX, 10 pmol/100 nL/5 minutes, Ascent Scientific, Bristol, UK or Sigma-Aldrich) were delivered by a pump (Picopump 1400 pressure ejector, World Precision Instruments, Sarasota, FL). ACSF vehicle was composed of (in mM): NaCl 148, KCl 3, CaCl_2_ 1.4, MgCl_2_ 0.8, Na_2_HPO_4_ 1, D-glucose 20, pH 7.3 with NaOH (chemicals from Fisher Scientific).

#### Histology for *in vivo* experiments

At the conclusion of experiments, Pontamine Sky Blue (2%, Alfa Aesar) was ejected from the high impedence recording electrode (−30 µA, 20–30 min). Fast Green (2%, Sigma-Aldrich) was included in the infusion cannula and low impedence electrodes to mark their location. Rats were decapitated and their brains were removed and stored in 4% paraformaldehyde (Sigma-Aldrich) in 0.1 M phosphate-buffered saline (PBS) overnight, and then cryoprotected in 25% sucrose (Sigma-Aldrich) in 0.1 M PBS. Brains were sliced into 60 µm thick sections using a freezing microtome (Leica Microsystems Inc, Buffalo Grove, IL) and stained with cresyl violet. Recording, infusion and stimulation sites were verified under light microscopy and plotted based on a rat brain atlas^119^.

### Data analysis

When possible, an individual rat was used to measure both LFPs and single units. When drug was applied locally by pressure ejection during LFP recordings, PTX and ACSF vehicle were applied to opposite hemispheres of the same rat in a counterbalanced manner. Multiple different manipulations were not performed in the same hemisphere.

#### Analysis of extracellular recordings

BLA contains projection neurons and interneurons. Putative projection neurons and interneurons were separated based on previously-established criteria that utilize action potential width^58,118,120^. However, because these characteristics can vary depending on electrode and filter settings, these criteria were retested by measuring the half-width of action potentials and plotting a frequency distribution of half-widths. The best-fit for this distribution was tested between one 2^nd^ order polynomial (indicative of a single population) and two 2^nd^ order polynomials (indicative of two populations). When data fits are consistent with two populations, a cut-off of 0.225 ms appropriately separates the populations under these recording and filtering conditions^58^. To reduce uncertainty, a buffer on either side of this intersection (0.205–0.240 ms) was added, and neurons within this window were excluded. Neurons above this cut-off were classified as projection neurons, while neurons below this cut-off were classified as putative interneurons. To increase reliability, only neurons that showed a biphasic action potential waveform were included.

Projection neurons were included in analysis if they met the following criteria: they were located within the BLA as determined by reconstruction based on histological staining, action potentials had a clearly visible signal to noise ratio (>3:1), the firing rate was stable, and the action half–width was greater than 0.240 ms. The spontaneous firing rate was measured as the number of action potentials/s (Hz) over a minimum of 4 minutes.

#### Single unit response to PFC stimulation

For analysis of response to PFC stimulation, the action potential firing over a 3 sec peri-stimulation period (40–100 repetitions) at the same stimulation intensities were organized into peri-stimulus time histograms (PSTHs), divided into 300 bins (10 msec bin width) and the number of action potentials in each bin was tabulated. The response of BLA projection neurons to PFC stimulation was then classified into one of 3 qualitative types, based on the firing changes after stimulation: Inhibitory response, Excitatory response and No response. Each neuron was characterized and grouped by response type. After categorization by type, the response to PFC stimulation was averaged within each group and compared between adolescent and adults or between intra-BLA infusion groups.

Inhibitory response: Neurons were grouped in the Inhibitory response type if they displayed suppressed firing after PFC stimulation compared to null stimulation (0 mA). This was defined as 3 consecutive bins with a firing rate that was ≥2 standard deviations lower than the average null stimulation firing or 5 consecutive bins with 0 action potentials during a 1 s time window after stimulation. The first of these bins was marked as the onset of inhibition. Area under PSTH was used to quantify the degree of inhibition. Area under the PSTH produces a measure of inhibition that incorporates a temporal component. The response was normalized to the baseline firing rate [(AP_post_ − AP_pre_) ÷ AP_pre_], where AP_pre_ = mean firing rate of the pre-stimulation epoch and AP_post_ = mean firing rate of each 10 ms bin during a 300 ms window immediately following stimulation. This normalized response at each bin was summated across the 300 ms window. The duration of inhibition was also measured. The time from the inhibition onset was first determined (as above, derived from averaged data). The time from this onset of inhibition to the time of the first action potential after inhibition onset was measured during each PFC stimulation sweep. These time epochs were averaged for each neuron as the measure for duration of inhibition.

Excitatory response: Neurons were grouped in the Excitatory response type if they displayed an increase greater than 5 times baseline firing rate within a 10–30 ms window after the stimulation, and the response was consistent with a monosynaptic input. Thus, neurons were excluded if the apparent excitatory effect fit criteria for antidromic responses (<1 ms variability of latency and reliably followed 300 Hz stimulation) or a polysynaptic response (latency >30 ms, and variability of response >5 ms). The excitatory response was measured as the action potential firing probability in the 10–30 msec window after stimulation after mean baseline firing rate was subtracted.

No response: Neurons were grouped in the No response type if they fell short of the criteria described above.

#### LFP response to PFC stimulation

Traces were filtered (200 Hz) and averaged (at least 10 consecutive traces). The slope of the evoked LFP was determined by measurement of the rise/run using Axograph X software (Sydney, Australia). The slope of each LFP evoked during a train (10 stimuli) was measured. LFP facilitation/depression was normalized and quantified as [slope LFP_x_ ÷ slope LFP_1_], where LFP_x_ = the slope of each LFP during a train, and LFP_1_ = slope of the 1^st^ LFP of the train. The summation ratio was quantified as the slope of the last LFP (10^th^ LFP) normalized to the first LFP of the train (LFP_10_ ÷ LFP_1_). A ratio >1 indicates *summation*, a ratio <1 indicates *suppression*.

The data analysed during the current study are available from the corresponding author on reasonable request.

### Statistical analysis

Statistical tests were performed using Prism 5 software (GraphPad, La Jolla, CA). A p value < 0.05 was considered statistically significant. The proportion of neurons exhibiting each of the 3 types of responses to PFC stimulation was compared between groups using a Chi–square test. Data were tested for normal distribution (D’Agostino and Pearson normality test). When two groups were subjected to a planned comparison they were compared with a two-tailed unpaired t-test. When multiple factors were analyzed, measures were compared using a two–way ANOVA with age (adolescent or adult) as a main factor and stimulation intensity or stimulation pulse number as a repeated measures factors when appropriate. Holm-Sidak’s multiple comparisons test was used for further comparison when a significant difference was found in ANOVA. All data were presented as mean ± SEM, unless otherwise specified.

## Electronic supplementary material

Supplementary Figures
