# Supplementary material for: Limited prefrontal cortical regulation over the basolateral amygdala in adolescent rats
Source: Sci Rep. 2018 Nov 21;8:17171. doi: 10.1038/s41598-018-35649-0 (PMC6249319; doi:10.1038/s41598-018-35649-0)
Supplement: Supplementary file 1 — Supplementary Figures [file 41598_2018_35649_MOESM1_ESM.pdf]

Limited prefrontal cortical regulation over the basolateral amygdala in adolescent rats

Ryan Selleck, Wei Zhang, Hannah Mercier, Mallika Padival and J. Amiel Rosenkranz

Supplementary Figure legends

Supplementary Figure 1. Local administration of picrotoxin (PTX) or vehicle (aCSF). Infusions of either PTX or aCSF were made in one hemisphere (hemi.). In approximately half of the animals, both hemispheres were infused, in a counterbalanced manner. **(A)** Sites of local administration were confirmed by the presence of Fast Green after histological processing. Shown here is an example of a Fast Green deposit in lightly Nissl-stained tissue, with an overlay of the BLA boundaries from an atlas, at 5x magnification and **(B)**10x magnification. **(C)** The first set of experiments included histologically verified LFP recording sites from the LAT (n=11 hemispheres from 11 rats) and BA (n=11 hemispheres from 11 rats). **(D)** Sites for local administration of PTX and vehicle were histologically verified to be within the LAT or BA (n=5 hemispheres, 5 rats/group). The numbers adjacent to each atlas section are the distance (mm) from bregma. Modified from Paxinos and Watson, 2007, with permission from Elsevier, copyright owner.

Supplementary Figure 2. PFC stimulation sites. The site of PFC stimulation was verified histologically from Nissl stained tissue. The site was determined to be in PrL or IL based on the tip of the stimulation electrode mark. **(A)** An example of a histologically-verified stimulation site in the PFC at 5x (top) and 10x (bottom) with an overlay of the PFC boundaries from an atlas. **(B-D)** The number of rats used for each study is labeled. The numbers adjacent to each atlas section are the distance (mm) from bregma. Both hemispheres were examined in most recordings, therefore the numbers of hemispheres and rats are listed. Modified from Paxinos and Watson, 2007, with permission from Elsevier, copyright owner.

Supplementary Figure 3. LFPs in the LAT and BA nuclei evoked by trains of PFC stimulation in adolescent rats. (A) Train stimulation at 10, 20, or 40 Hz was applied to PFC and LFPs were measured in the adolescent (PND 39) BLA. **(A)** The recorded LFPs in LAT after administration of vehicle (left) or PTX (right). **(B)** The recorded LFPs in BA after administration of vehicle (left) or PTX (right).

Supplementary Figure 4. LFPs in the LAT and BA nuclei evoked by trains of PFC stimulation in adult rats. (A) Train stimulation at 10, 20, or 40 Hz was applied to PFC and LFPs were measured in the adult (PND 72-75) BLA. **(A)** The recorded LFPs in LAT after administration of vehicle (left) or PTX (right). **(B)** The recorded LFPs in BA after administration of vehicle (left) or PTX (right).

Supplementary Figure 5. Single neurons were recorded throughout the BLA. The final recording site was marked with Pontamine dye. **(A)** The Pontamine dye (arrow) was observed in Nissl stained tissue and recording sites were reconstructed at 5x and **(B)**

10x magnification with an overlay of the BLA boundaries from an atlas. (C) The verified recording sites were mapped as either LAT or BA in adult (PND 72-75) rats **(C)** and adolescent (PND 39) rats **(D)**. The number of neurons from each condition are listed below the maps. Both hemispheres were examined in most recordings, therefore the numbers of hemispheres and rats are listed. The numbers adjacent to each atlas section are the distance (mm) from bregma. Modified from Paxinos and Watson, 2007, with permission from Elsevier, copyright owner.

Additional references
Paxinos, G. & Watson, C. The Rat Brain in Stereotaxic Coordinates, Sixth Edition (Academic Press, 2007).
Atlas panels modified from Paxinos and watson, 2007. Permission for reuse granted by copyright holder, Elsevier.

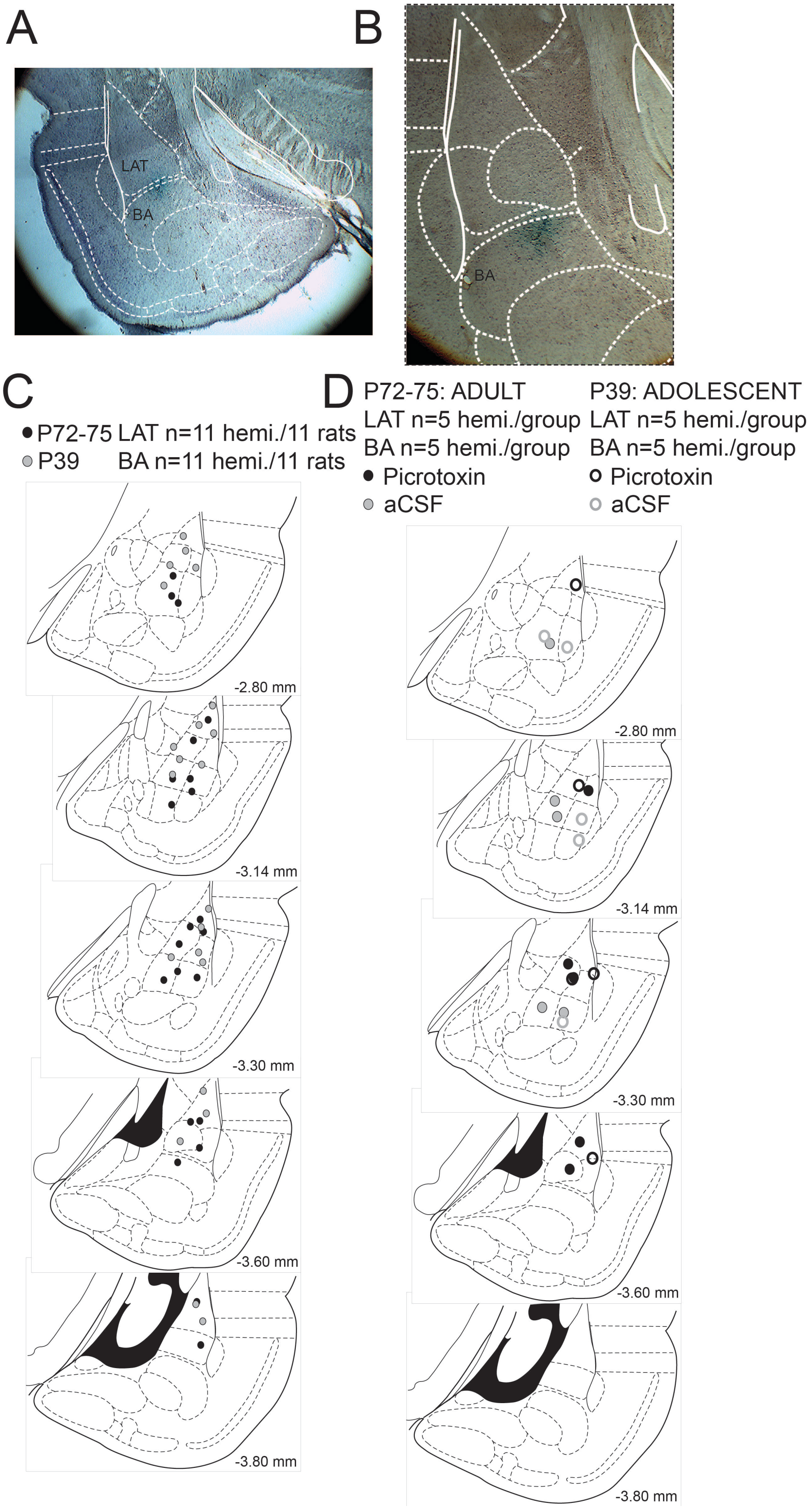
A
LAT
BA
B
BA
C
P72-75 LAT n=11 hemi./11 rats
P39 BA n=11 hemi./11 rats
-2.80 mm
-3.14 mm
-3.30 mm
-3.60 mm
-3.80 mm
D
P72-75: ADULT
LAT n=5 hemi./group
BA n=5 hemi./group
Picrotoxin
aCSF
P39: ADOLESCENT
LAT n=5 hemi./group
BA n=5 hemi./group
Picrotoxin
aCSF
-2.80 mm
-3.14 mm
-3.30 mm
-3.60 mm
-3.80 mm

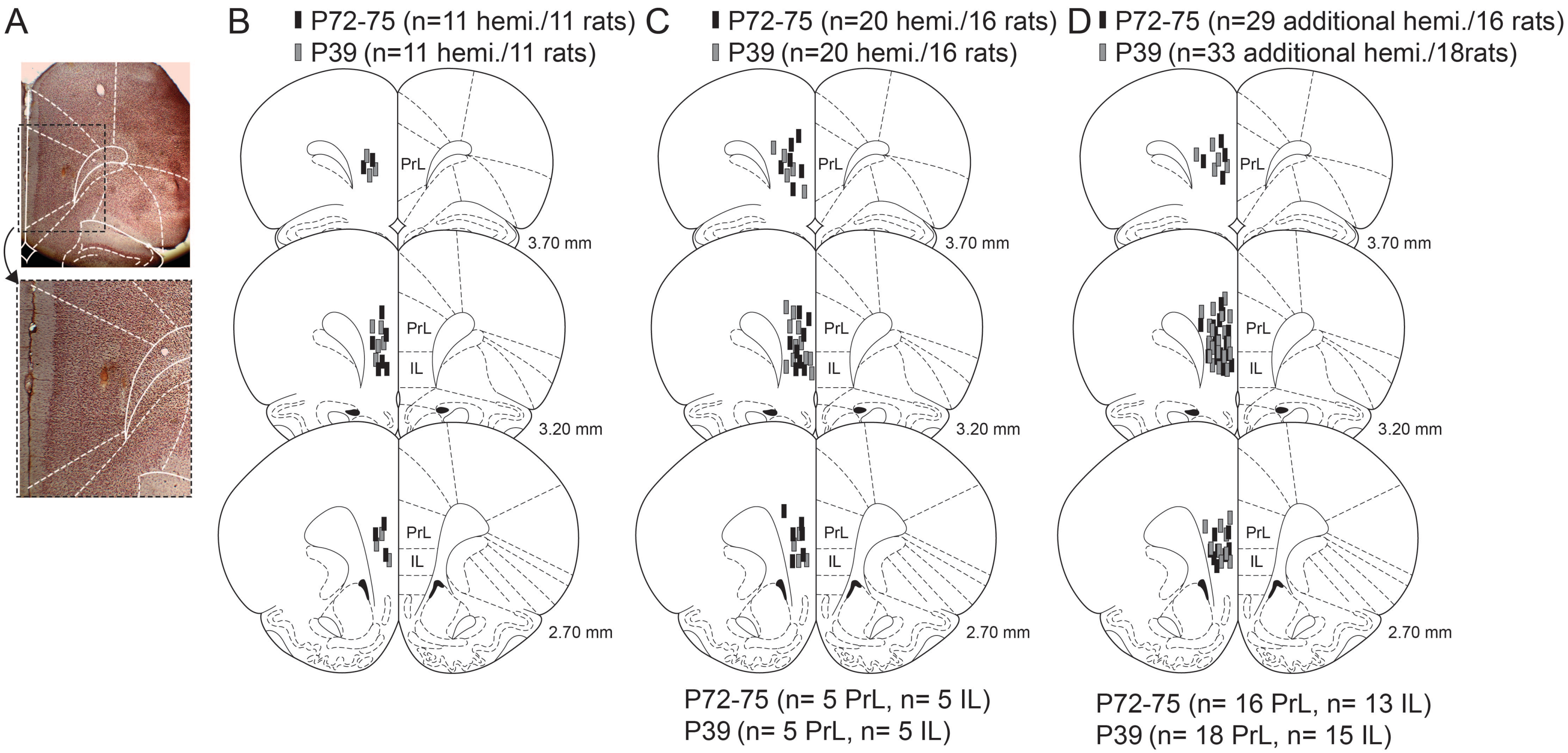
A
B
P72-75 (n=11 hemi./11 rats)
P39 (n=11 hemi./11 rats)
C
P72-75 (n=20 hemi./16 rats)
P39 (n=20 hemi./16 rats)
D
P72-75 (n=29 additional hemi./16 rats)
P39 (n=33 additional hemi./18rats)
PrL
IL
3.70 mm
3.20 mm
2.70 mm
P72-75 (n= 5 PrL, n= 5 IL)
P39 (n= 5 PrL, n= 5 IL)
P72-75 (n= 16 PrL, n= 13 IL)
P39 (n= 18 PrL, n= 15 IL)

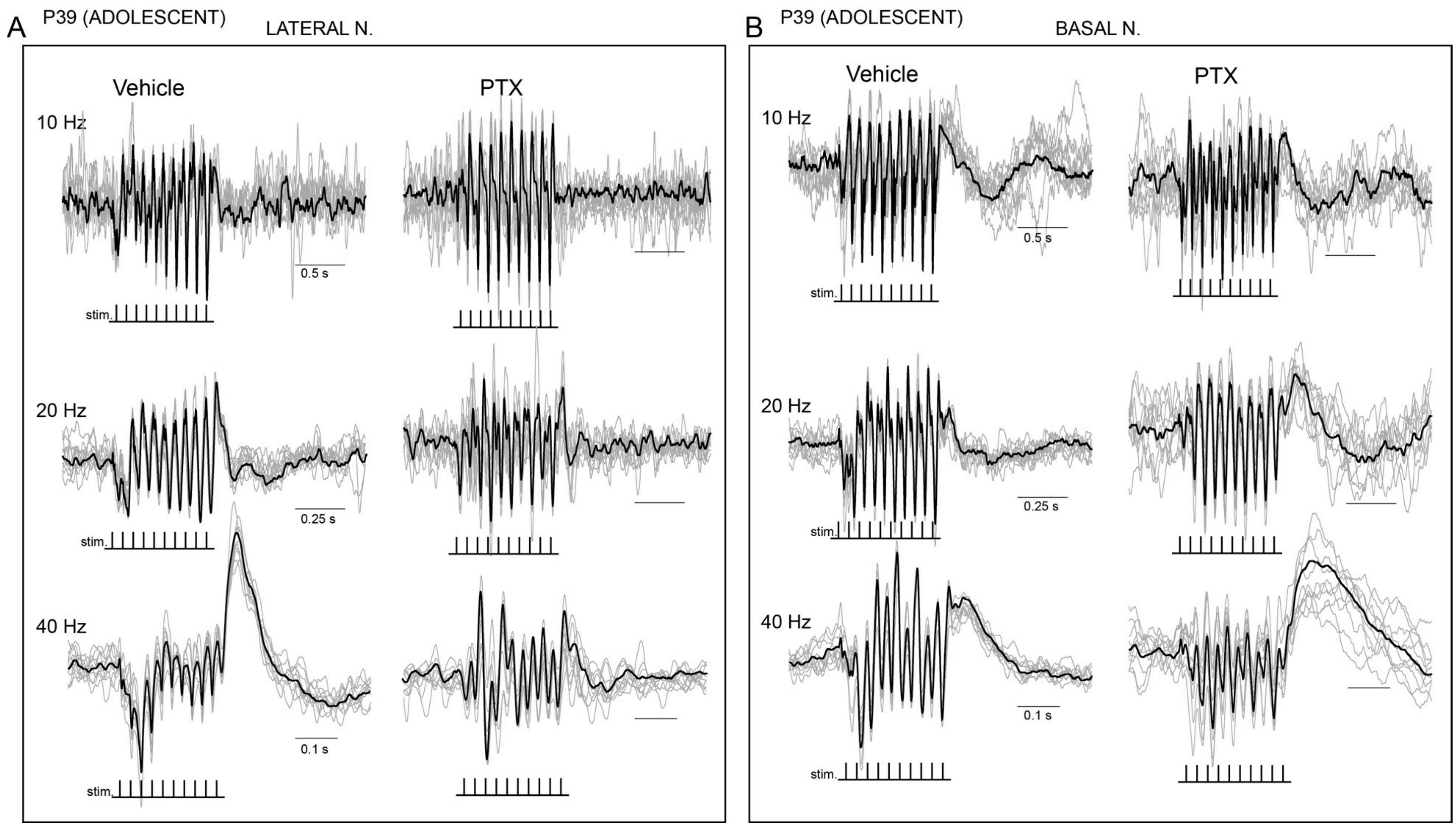
A
P39 (ADOLESCENT)
LATERAL N.
Vehicle
PTX
10 Hz
20 Hz
40 Hz
stim.
0.5 s
0.25 s
0.1 s
B
P39 (ADOLESCENT)
BASAL N.
Vehicle
PTX
10 Hz
20 Hz
40 Hz
stim.
0.5 s
0.25 s
0.1 s

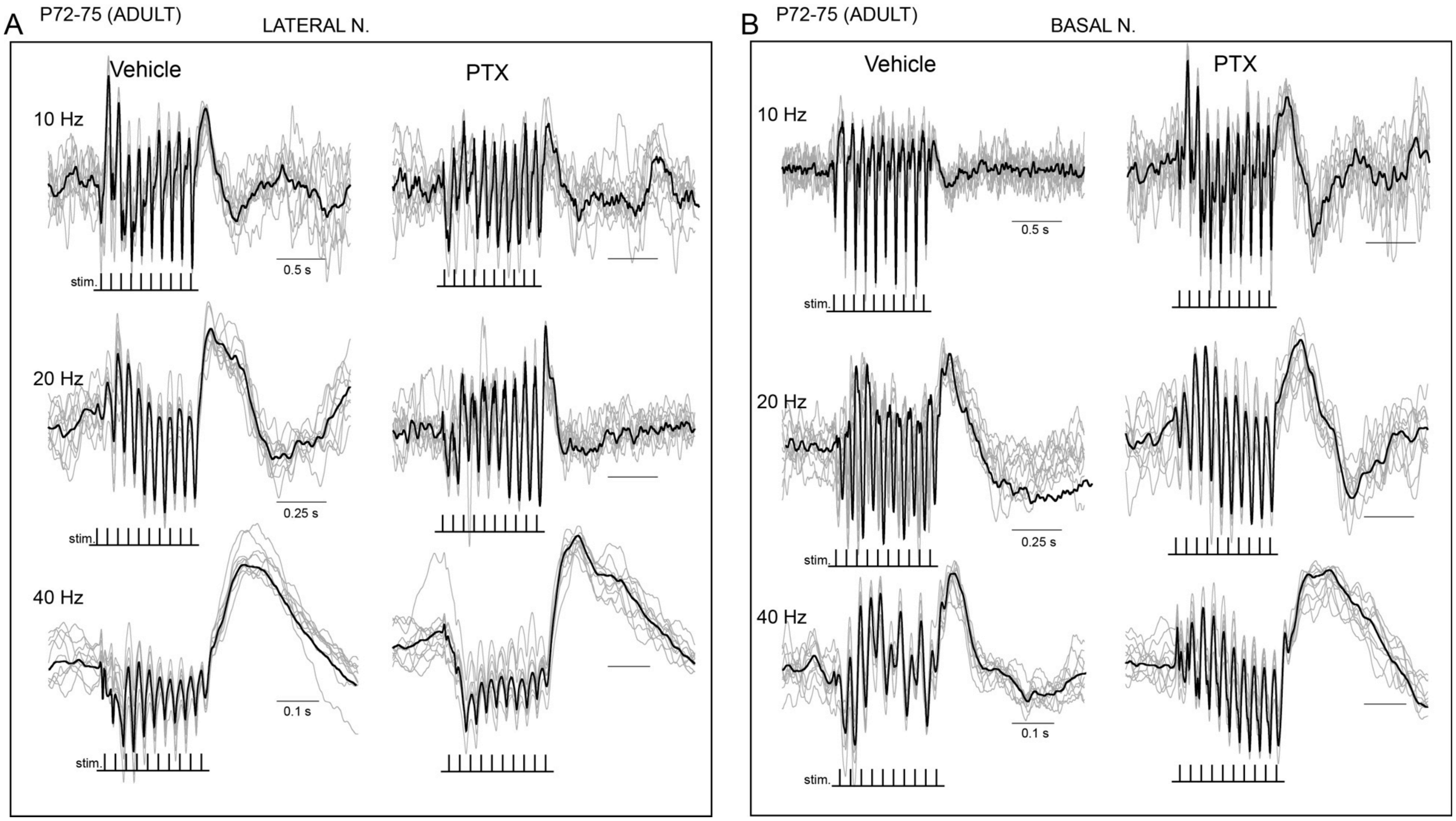
A
P72-75 (ADULT)
LATERAL N.
Vehicle
PTX
10 Hz
20 Hz
40 Hz
stim.
0.5 s
0.25 s
0.1 s
B
P72-75 (ADULT)
BASAL N.
Vehicle
PTX
10 Hz
20 Hz
40 Hz
stim.
0.5 s
0.25 s
0.1 s

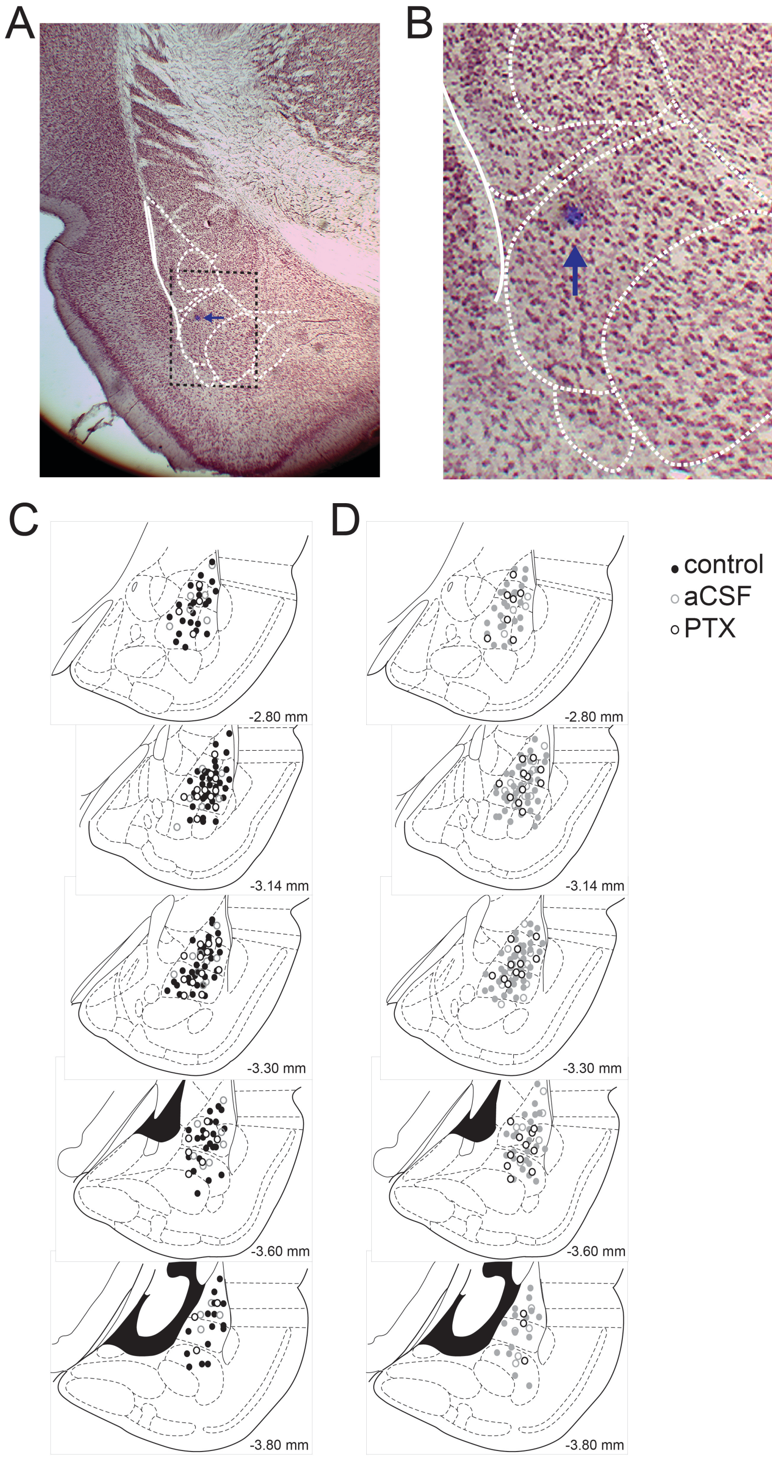

ADULT (P72-75)

PFC stim - N=49 hemi./25 rats

control: 27 hemi.

LAT n=67 neurons

BA n=56 neurons

vehicle:11 hemi.

LAT n=21 neurons

BA n=20 neurons

PTX: 11 hemi.

LAT n=18 neurons

BA n=21 neurons

ADOLESCENT (P39)

PFC stim - N=53 hemi/28 rats

control: 29 hemi.

LAT n=69 neurons

BA n=71 neurons

vehicle: 12 hemi.

LAT n=23 neurons

BA n=26 neurons

PTX: 12 hemi.

LAT n=24 neurons

BA n=22 neurons
